# 294. Risk factors of COVID-19 associated pulmonary aspergillosis in a high endemic setting and development of a bedside clinical risk prediction score.

**DOI:** 10.1093/ofid/ofac492.372

**Published:** 2022-12-15

**Authors:** Merlin Moni, Teny M M John, Abdul Razak Moosa, Kiran G Kulirankal, Fabia Edathadathil, T S Dipu

**Affiliations:** Amrita Institute of Medical Sciences, Kochi, Kochi, Kerala, India; M D Anderson Cancer Centre, houston, Texas; Amrita Institute of Medical Sciences, Kochi, Kerala, India; Amrita Institute of Medical Sciences and Research Center, kochi, Kerala, India; Amrita Institute of Medical Sciences and Research Center, kochi, Kerala, India; Amrita Institute of Medical Sciences and Research Center, kochi, Kerala, India

## Abstract

**Background:**

India has a high burden of invasive fungal infections. Although invasive aspergillosis was also reported during the COVID-19 pandemic, the real world data on the risk factors and outcome of CAPA are limited. Our aim is to determine risk factors and clinical outcomes of CAPA and develop a prediction model for patient stratification.

**Methods:**

A retrospective, case-control study was conducted at a 1300-bedded South Indian tertiary care academic centre from June 1st, 2020 to May 31st, 2021. CAPA cases were defined by 2020 ECMM/ISHAM consensus criteria as possible, probable, and proven infection. Age and admission period matched control group with COVID-19 but without aspergillosis was selected in a 1:1 ratio. A risk scoring stratification for CAPA was developed based on the significant CAPA risk factors using logistic regression model.

**Figure 1:**
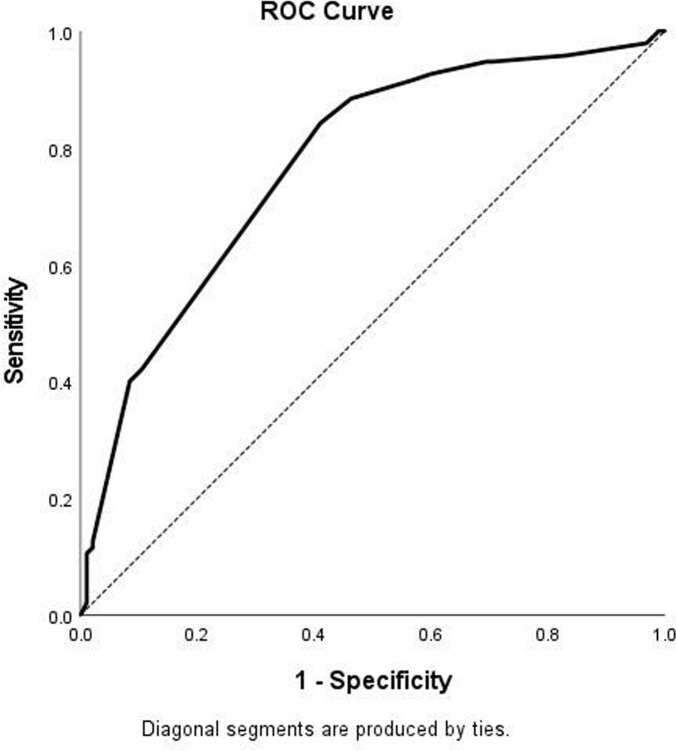
Receiving operating characteristic curve of CAPA incidence score for predicting CAPA in the study cohort

**Results:**

95 CAPA cases, of which 75(79%) were probable and 20(21%) possible, were diagnosed during the study period. 84(88.4%) patients had moderate to severe COVID-19, and 75(78.9%) were treated with steroids, most commonly dexamethasone (Table 1). The time from COVID-19 diagnosis to CAPA was 13 days (IQR 12). 40(42.1%) of patients were on mechanical ventilation at CAPA diagnosis. Outcome measures (MV, NIV and hospital/ICU stay) were significantly higher in CAPA patients compared to controls(Table 2). Neutropenia, use of steroids, broad spectrum antibiotic use, fluconazole prophylaxis and absence of co-infecting pathogen were found to be significant factors associated with CAPA(p< 0.05) (Table 3). An optimal risk score of ≥10.00 predicted CAPA with a sensitivity of 84.2% and a specificity of 59% with an area under the curve of 0.77 (PPV=67.23%, NPV=78.87%)(Fig 1). 75(78.9%) patients had positive serum aspergillus galactomannan with an average value of 1.89±1.65. 28-day (41.1% vs 33.7%, p=0.13) and 6-week all cause-mortality (48.4% vs 37.9%, p=0.07) were higher, but not statistically significant, for CAPA.

**Table 1:**
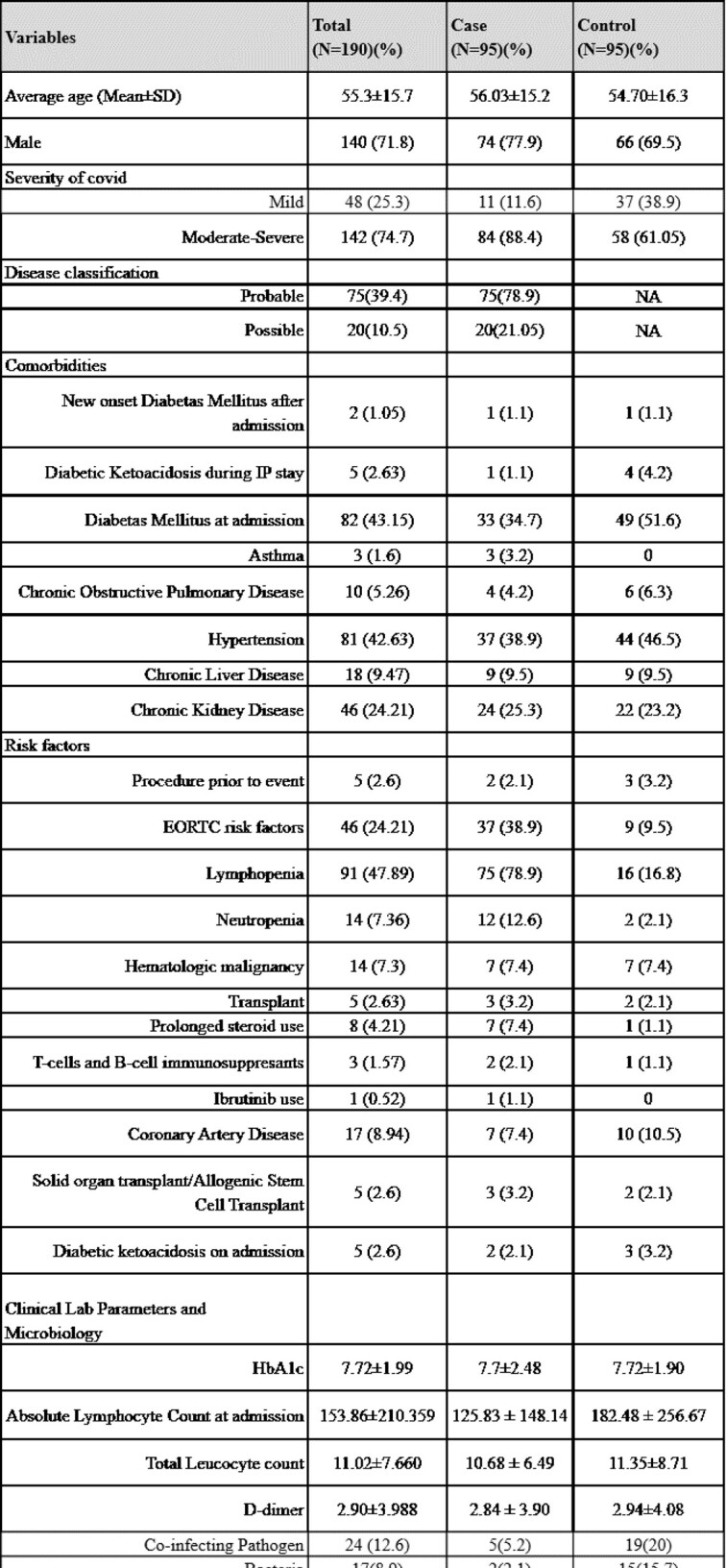
Baseline characteristics and risk factors

**Table 2:**
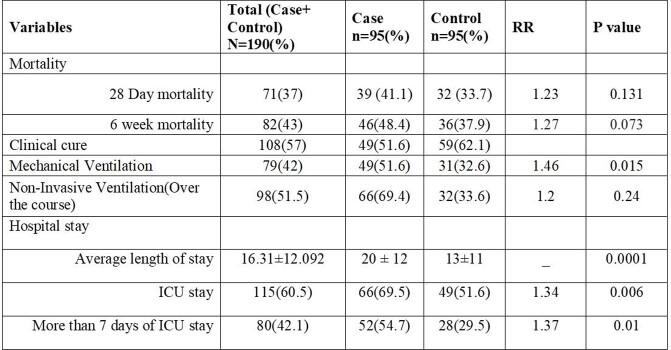
Primary and secondary outcomes

**Table 3:**
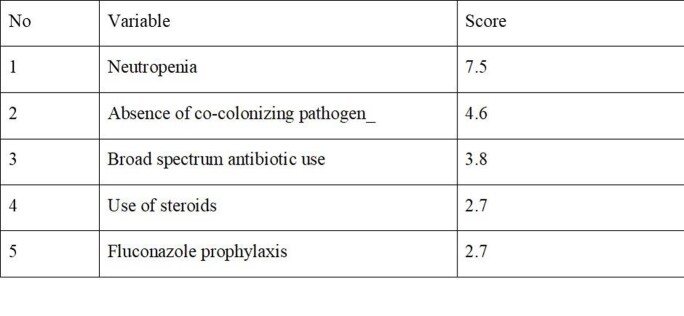
CAPA incidence scoring table

Significant univariate variables were included in the multivariate logistic regression model and predicted probabilities based on the beta co-efficients of the significant variables in the model were transformed to generate CAPA incidence score for patients.

**Conclusion:**

Risk factors of CAPA in Indian were similar to those reported previously in other countries. CAPA can be seen in severe COVID-19 patients who are not mechanically ventilated. A CAPA risk scoring system, that needs external validation, is a simple and feasible tool that could be useful in stratification of patients suspected of CAPA.

**Disclosures:**

**All Authors**: No reported disclosures.

